# An inhibitor-driven study for enhancing the selectivity of indirubin derivatives towards leishmanial Glycogen Synthase Kinase-3 over leishmanial cdc2-related protein kinase 3

**DOI:** 10.1186/1756-3305-7-234

**Published:** 2014-05-20

**Authors:** Antonia Efstathiou, Nicolas Gaboriaud-Kolar, Despina Smirlis, Vassilios Myrianthopoulos, Konstantina Vougogiannopoulou, Alexandros Alexandratos, Marina Kritsanida, Emmanuel Mikros, Ketty Soteriadou, Alexios-Leandros Skaltsounis

**Affiliations:** 1 Laboratory of Molecular Parasitology, Department of Microbiology, Hellenic Pasteur Institute, 127 Vas.Sofias Ave, 11521 Athens, Greece; 2 Laboratories of Pharmacognosy and Pharmaceutical Chemistry, Department of Pharmacy, University of Athens, Panepistimiopolis-Zografou, 15771 Athens, Greece; 3 Department of Pharmacognosy and Natural Product Chemistry, Faculty of Pharmacy, National and Kapodistrian University of Athens, Athens, Greece

**Keywords:** *Leishmania*, Indirubins, *L*GSK-3, Szmap, Binding site solvation

## Abstract

**Background:**

In search of new antiparasitic agents for overcoming the limitations of current leishmaniasis chemotherapy, we have previously shown that 6-bromoindirubin-3*'*-oxime (6BIO) and several other 6-substituted analogues of indirubin, a naturally occurring bis-indole present in mollusks and plants, displayed reverse selectivity from the respective mammalian kinases, targeting more potently the leishmanial Cyclin-Dependent Kinase-1 (CDK1) homologue [cdc2-related protein kinase 3 (*L*CRK3)] over leishmanial Glycogen Synthase Kinase-3 (*L*GSK-3). This reversal of selectivity in *Leishmania* parasites compared to mammalian cells makes the design of specific indirubin-based *L*GSK-3 inhibitors difficult. In this context, the identification of compounds bearing specific substitutions that shift indirubin inhibition towards *L*GSK-3, previously found to be a potential drug target, over *L*CRK3 is imperative for antileishmanial targeted drug discovery.

**Methods:**

A new in-house indirubin library, composed of 35 compounds, initially designed to target mammalian kinases (CDKs, GSK-3), was tested against *Leishmania donovani* promastigotes and intracellular amastigotes using the Alamar blue assay. Indirubins with antileishmanial activity were tested against *L*GSK-3 and *L*CRK3 kinases, purified from homologous expression systems. Flow cytometry (FACS) was used to measure the DNA content for cell-cycle analysis and the mode of cell death. Comparative structural analysis of the involved kinases was then performed using the Szmap algorithm.

**Results:**

We have identified 7 new indirubin analogues that are selective inhibitors of *L*GSK-3 over *L*CRK3. These new inhibitors were also found to display potent antileishmanial activity with GI_50_ values of <1.5 μΜ. Surprisingly, all the compounds that displayed enhanced selectivity towards *L*GSK-3, were 6BIO analogues bearing an additional 3*'*-bulky amino substitution, namely a piperazine or pyrrolidine ring. A comparative structural analysis of the two aforementioned leishmanial kinases was subsequently undertaken to explain and rationalize the selectivity trend determined by the *in vitro* binding assays. Interestingly, the latter analysis showed that selectivity could be correlated with differences in kinase solvation thermo dynamics induced by minor sequence variations of the otherwise highly similar ATP binding pockets.

**Conclusions:**

In conclusion, 3*'*-bulky amino substituted 6-BIO derivatives, which demonstrate enhanced specificity towards *L*GSK-3, represent a new scaffold for targeted drug development to treat leishmaniasis.

## Background

Leishmaniasis is a vector-borne neglected tropical disease caused by parasitic protozoa of the genus *Leishmania*[1]. Mammals, including humans, are infected by *Leishmania* promastigote parasites via the bite of female phlebotomine sand flies [2,3]. After *Leishmania* promastigotes pass into the mammalian blood, they are phagocytosed by macrophages and once located within the macrophages’ phagolysosomes, promastigotes transform into the non-motile amastigote form and multiply [4]. More than 350 million people are at risk of *Leishmania* infection and about 1.5-2 million new cases and 500,000 deaths are considered to occur every year in the endemic areas [1]. The increasing resistance of *Leishmania* parasites and the toxicity of the current therapy as well as the non-existence of a human vaccine, generate an urgent need to discover effective, new-targeted drugs for treating leishmaniasis [5,6]. Research on natural products has been proved to be promising for discovering new lead structures in a variety of diseases including leishmaniasis [6]. Amongst natural product scaffolds, alkaloids display considerable structure diversity that can be exploited for the discovery of novel antileishmanials [6]. Moreover, marine indole-based alkaloid scaffolds [7] like variolin [8], roscovitine [9], leucettines [10] and halogenated indirubins [11], known to target kinases, represent a significantly large pool of compounds for the discovery of new targeted antileishmanial treatment [12,13].

Specifically, indirubin is a naturally occurring bis-indole found in different species like indigo-bearing plants (*Isatis spp.*, *Polygonum spp.*) or marine organisms (Murex shellfish family, *Hexaplex trunculus*) [11]. It has been identified as a potent inhibitor of protein kinases (CDKs) [14], whereas naturally halogenated indirubins such as 6-bromoindirubin (6BI) demonstrate selective inhibitory activity towards the mammalian Glycogen-Synthase Kinase-3 (GSK-3) [15]. The semi-synthetic analogue of 6BI, 6-bromoindirubin-3′-oxime (6BIO) was developed as a potent and selective GSK-3β inhibitor and is currently considered as one prototype inhibitor of that mammalian kinase [16].

The Cyclin-Dependent Kinase1 (CDK1) homologue of *Leishmania* [cdc2-related protein kinase 3 (*L*CRK3)] and the glycogen synthase kinase-3short (*L*GSK-3) have emerged amongst other kinases as putative molecular drug targets for the treatment of leishmaniasis [13,17-20]. *L*CRK3 was characterized by genetic analyses as an essential protein for parasite viability and a central regulator of cell-cycle progression [21]. Inhibition of *L*CRK3 in *Leishmania* parasites resulted in a G2/M cell-cycle arrest, which was subsequently followed by an apoptosis-like death of the parasites [13,21]. Recently, the trypanosomatid GSK-3 was identified as a potential drug target for treatment of parasitic diseases [13,19]. In a previous study, we showed that *L*GSK-3 was essential for parasitic viability, and its inhibition caused cell-cycle defects and apoptosis-like death [13]. We have previously shown that 6-substituted indirubin analogues, including 6BIO, inhibit more potently *L*CRK3 than *L*GSK-3, a reverse selectivity compared to the corresponding mammalian homologues. The only exceptions to this reversal were the bisubstituted 5-methyl-6-bromoindirubin-3′-oxime (5-Me-6BIO) which was 7-fold more selective towards *L*GSK-3 over *L*CRK3 and 6-bromoindirubin-3′-acetoxime (6-BIA) which inhibited both kinases equally well [13]. This reversal indicated that the mammalian GSK-3/CDK1 selectivity determinants differ from the corresponding parasitic ones. Herein, we describe the evaluation of indirubin analogues, initially designed to target mammalian kinases, against *Leishmania* parasites. One main objective of this study was to improve indirubin selectivity towards *L*GSK-3 over *L*CRK3. This knowledge is important for antileishmanial drug discovery since previous studies showed that *L*CRK3 inhibition did not translate very well into antiparasitic activity [18,22]. In addition, this knowledge is anticipated to be crucial for Structure Activity Relationship studies (SARs) and modification of the lead compound to more potent and specific inhibitor. Overall, this approach led to the identification of indirubin based antileishmanial agents favoring inhibition of *L*GSK-3 over *L*CRK3. Interestingly, the compounds with *L*GSK-3 specificity were 6BIO derivatives possessing bulky amino substitution namely a piperazine or pyrrolidine ring at position 3′, suggesting that these substitutions increase the affinity of the compound towards *L*GSK-3 but not towards *L*CRK3. A comparative structural analysis of the two aforementioned parasitic kinases was undertaken as a means to explain and rationalize the selectivity trend determined by *in vitro* assays, showing that the enhanced selectivity of 6-bromo-3′-substituted indirubins for *L*GSK-3 can be attributed to differences in the solvation pattern of the otherwise similar ATP binding pocket.

## Methods

### Chemical synthesis

The studied compounds have been synthesized according to the methodology developed previously [11,16,23,24].

### Evaluation of the antileishmanial activity of indirubins against *L. donovani* promastigotes and intracellular amastigotes *in vitro*

All compounds were dissolved in DMSO at 10 mM and serial dilutions in DMSO were made (1 mM and 100 μM). The analogues were further diluted in the culture medium to give the desired final concentrations.

The antileishmanial activity was determined using the Alamar blue assay [25]. *L. donovani* promastigotes (MHOM/ET/0000/HUSSEN) which were frequently passed in BALB/c mice [26] were used in all experiments. Specifically, 2.5×10^6^ cells/ml of *L. donovani* promastigotes in the stationary phase were seeded into 96-well flat bottom plates in total volume of 200 μl M199 without phenol red per well. In triplicates, indirubins were added in increasing concentrations and equivalent volumes of the solvent DMSO (<0.1%v/v) were used as control. After incubation of the parasites for 72 hrs at 26°C, Alamar blue (20 μl/well) was added for a further 24 hrs and colorimetric changes were read at 550 nm with reference wavelength 620 nm. Calculation of the compound concentration that induces 50% reduction of the growth rate of the promastigotes (GI_50_ values for 50% growth inhibition) was performed using the parasites treated with DMSO as control growth rate sample. GI_50_ values were determined from dose–response curves via linear interpolation.

For the *in vitro* infection evaluation of indirubins’ antileishmanial activity, 2×10^5^ J774.1 cell line macrophages per ml in 200 μl RPMI supplemented with 10% (v/v) HIFBS (heat-inactivated fetal bovine serum), 10 mM HEPES and penicillin-streptomycin (final concentration 100U ml^−1^), were seeded into 96-well flat bottom plates. The macrophages were left to adhere overnight at 37°C in an atmosphere of 5% CO_2_. Afterwards, the macrophage infection was performed at a ratio of 10 parasites/macrophage for 24 hrs at 37°C in 5% CO_2_, followed by the incubation of the infected macrophages with the indirubins for 72 hrs. DMSO-treated macrophages, which were infected with parasites, were used as controls. After this 72 hrs period and the removal of the medium, the macrophages were lysed with 100 μl 0.01% (v/v) SDS in PBS for 30 min at 37°C. Then, 100 μl Schneider’s medium was added to each well and amastigote growth was assessed by the addition of Alamar blue (20 μl/well) and the plates were incubated for 48 hrs at 37°C [27]. Calculation of the GI_50_ values was performed as previously described [13].

In order to confirm the *in vitro* infection evaluation results of indirubins’ antileishmanial activity, we also performed the assay with 2×10^5^ peritoneal macrophages, collected from BALB/c mice (4–6 weeks old), 72 hrs after the intraperitoneally administration of 1 ml sterile thioglycollate medium (4% w/v, Becton Dickinson, Sparks, MD, USA). The mice, which were used with prior approval by the Animal Bioethics Committee of the Hellenic Pasteur Institute (HPI; Athens, Greece) according to the Directive 2010/63/EE of the council of Europe, for the protection of vertebrates/animals, were euthanized for the recovery of peritoneal macrophages. The peritoneal macrophages were centrifuged (1,200 rpm, 4°C, 10 min) and washed 3 times with RPMI-1640 medium. After the collection of the peritoneal macrophages the steps followed were the same as the ones described above for the *in vitro* infection assay with J774.1 cell line macrophages.

For the compounds **11–17**, the intracellular amastigote assay was performed at first with both murine macrophagic cell-line J774.1 and peritoneal macrophages extracted from BALB/c mice. The results from both assays were significantly similar (data not shown) and therefore the following experiments were performed with J774.1 murine macrophages.

### Cell-cycle and cell-death analysis of indirubin-treated promastigotes by flow cytometry

*L. donovani* promastigotes in the stationary phase (2×10^7^cells/ml) were seeded at 10^6^cells/ml in M199 medium and incubated for 48 hrs, at 26°C with the GI_50_ concentration of each analogue or with DMSO, as control. For the analysis of the cell cycle, the procedure described in Georgopoulou *et al.*[28], was followed to prepare the samples for fluorescence-activated cell sorting. In order to determine the amount of cells, which display membrane fluidity perturbations, FACS analysis was performed using AnnexinV-FITC and propidium iodide (PI) staining (Apoptosis Detection kit, R & D Systems). All samples were analyzed using a Becton Dickinson FACS Calibur flow cytometer and data were analyzed using the Cell Quest software. All experiments were performed in triplicates.

### Kinase assays

Recombinant *L*GSK-3 and *L*CRK3 were produced and purified from *L. donovani* over-expressing transfectants and transgenic *L. mexicana* promastigotes respectively, as previously described [13]. *L*CRK3 expressing *L. mexicana* parasites were a kind gift from Dr. K. Grant. The Kinase Luminescent Assay Kit (Promega) was used to perform the kinase assays, following the manufacturer’s instructions as previously described [13]. For determining the kinase activity of *L*GSK-3, GS-1 peptide was used as a substrate (YRRAAVPPSPSLSRHSSPHQSpEDEEE) [29] while *L*CRK3 kinase assays were performed using histone H1 as a substrate [18], with increasing concentrations of each compound. IC_50_ values (μM) were determined from dose–response curves. All experiments were performed in triplicates.

### Computational analysis

Homology models were built for *L*GSK-3 and *L*CRK3 of three *Leishmania* species *(L. major, L. donovani, L. infantum-*Uniprot codes Q4QE15, A6N857, A4HXQ3, O96526, Q7K8Z1, A4ICT0*)* using Prime 3.1 and the corresponding cocrystal structures of human GSK-3 and CDK5 with indirubin (pdb codes 1UV5, 1UNH) as templates [30]. Computational analysis of protein solvation was performed using Szmap algorithm. Szmap implements a semi-continuous solvation model for mapping the surface of the protein and identifies hydration sites of positive (unstable) and negative (stable) free energy. Characterization of water molecules according to their free energy permits rational design of high affinity ligands, which either displace unstable waters or replace stable waters by polar groups of similar capability for accommodating electrostatic interactions with the protein.

## Results and discussion

In order to understand which specific substitutions on the indirubin analogues contribute to *L*GSK-3 selectivity, we evaluated a larger collection of indirubin analogues designed to target several mammalian kinases including CDKs and GSK-3, with pleiotropic substitutions in the indirubin backbone (Table 1). The indirubin scaffold was substituted with various functional groups, interventions initially designed for effective inhibition of mammalian GSK-3 [23]. Those 6BIO analogues bear bulky and hydrophilic amino chains in position 3′ aiming at the enhancement of the otherwise low indirubin water-solubility. To this end, we screened this set against *L. donovani* promastigotes (the insect form) and intracellular amastigotes (the form found in mammals) at 3 μM (Table 1). Their toxicity against the murine macrophagic cell-line J774.1 was assessed at 10 μΜ (Table 1). This initial screening showed that 9 out of 35 analogues, were able to induce 100% growth inhibition when tested against *L. donovani* promastigotes form at 3 μΜ. Both forms of the studied 6BIO analogues (free base and hydrochloride salt) were tested yet only one pair of compounds presented a small difference in activity (salt **11** was found active against *L. donovani* promastigotes compared to its corresponding base **10** which was inactive at 3 μΜ). This minor discrepancy can be attributed to differences in cell permeability between the two forms of the compound. Indeed, **10** is the only analogue possessing a non-substituted piperazine group and thus demonstrates a higher pKa (≈1 unit) value. This physicochemical parameter could have an effect on the overall solubility of **10** at the pH of culture media, resulting to the observed difference in activity. Such discrepancies between free base and corresponding salt forms have been described in previous studies reporting activity of antitrypanosomal agents against parasites [31]. Compounds **4** and **5** that were active against *L. donovani* promastigotes, with a GI_50_ of 1.15 ± 0.06 and 0.9 ± 0.11 respectively, were found to be toxic to the murine macrophagic cell-line J774.1 (GI_50_: 1.5 ± 0.07 and 1.5 ± 0.08 respectively). Thus, these compounds were not further studied for their activity against intracellular amastigotes. Subsequently, GI_50_ values (μM) of compounds **11–17** for both *L. donovani* promastigote and amastigote forms were determined.

**Table 1:** **Inhibitory activity of the studied indirubin analogues against** ***L. donovani*** **promastigotes and intracellular amastigotes**

								***L. donovani*** **(GI**_**50**_**μM)**		
**Cpd**	**Y**	**R**_**1**_	**R**_**2**_	**R**_**3**_	**R**_**4**_	**R**_**5**_	**R**_**6**_	**Promastigotes**	**Intracellular amastigotes**	**ΜΦ J774.1 (GI**_**50**_**μM)**
**1**	NO	H	Br	H	H	H		>3 μΜ	>3 μΜ	-
**2**	NO	H	Br	H	H	H		>3 μΜ	>3 μΜ	-
**3**	NO	H	Br	H	H	H		>3 μΜ	>3 μΜ	-
**4**	NO	H	Br	H	H	H		1.15 ± 0.06	-	1.5 ± 0.07
**5**	NO	H	Br	H	H	H		0.9 ± 0.11	-	1.5 ± 0.08
**6**	NO	H	Br	H	H	H		>3 μΜ	>3 μΜ	-
**7**	NO	H	Br	H	H	H		>3 μΜ	>3 μΜ	> 10
**8**	NO	H	Br	H	H	H		>3 μΜ	>3 μΜ	>10
**9**	NO	H	Br	H	H	H		>3 μΜ	>3 μΜ	>10
**10**	NO	H	Br	H	H	H		>3 μΜ	>3 μΜ	> 10
**11**	NO	H	Br	H	H	H		0.75 ± 0.05	0.59 ± 0.07	6.25 ± 0.08
**12**	NO	H	Br	H	H	H		0.65 ± 0.09	0.80 ± 0.12	2.60 ± 0.05
**13**	NO	H	Br	H	H	H		0.82 ± 0.21	0.85 ± 0.16	2.41 ± 0.11
**14**	NO	H	Br	H	H	H		1.40 ± 0.10	1.71 ± 0.09	9.20 ± 0.15
**15**	NO	H	Br	H	H	H		1.50 ± 0.14	2.44 ± 0.17	6.00 ± 0.08
**16**	NO	H	Br	H	H	H		0.76 ± 0.03	1.54 ± 0.11	5.93 ± 0.04
**17**	NO	H	Br	H	H	H		0.76 ± 0.04	1.22 ± 0.06	10.00 ± 0.02
**18**	NO	H	Br	H	H	H		>3 μΜ	>3 μΜ	-
**19**	NO	H	Br	H	H	H		>3 μΜ	>3 μΜ	-
**20**	O	H	H	Br	H	H	-	>3 μΜ	>3 μΜ	-
**21**	NO	H	H	Br	H	H	H	>3 μΜ	>3 μΜ	> 10
**22**	O	H	H	Br	COOH	H	-	>3 μΜ	>3 μΜ	-
**23**	NO	H	H	Br	COOH	H	H	>3 μΜ	>3 μΜ	-
**24**	O	H	H	Br	H	COOH	-	>3 μΜ	>3 μΜ	-
**25**	NO	H	H	Br	H	COOMe	H	>3 μΜ	>3 μΜ	-
**26**	NO	H	H	Br	H	COOH	H	>3 μΜ	>3 μΜ	-
**27**	NO	H	H	CF_3_	H	H	H	>3 μΜ	>3 μΜ	-
**28**	NO	H	H	CF_3_	COOMe	H	H	>3 μΜ	>3 μΜ	-
**29**	O	H	H	CF_3_	COOH	H	-	>3 μΜ	>3 μΜ	-
**30**	NO	H	H	CF_3_	COOH	H	H	>3 μΜ	>3 μΜ	-
**31**	O	H	H	CF_3_	H	COOH	-	>3 μΜ	>3 μΜ	-
**32**	NO	H	H	CF_3_	H	COOMe	H	>3 μΜ	>3 μΜ	> 10
**33**	NO	H	H	CF_3_	H	COOH	H	>3 μΜ	>3 μΜ	-
**34**	O	H	H	CF_3_		H	-	>3 μΜ	>3 μΜ	-
**35**	NO	H	H	CF_3_		H	H	>3 μΜ	>3 μΜ	-
**6BIO**	NO	H	Br	H	H	H	H	0.85 ± 0.05	0.70 ± 0.10	>25
**5-Me-6-BIO**	NO	CH_3_	Br	H	H	H	H	1.2 ± 0.2	1.0 ± 0.1	>25
**Amphotericin B**	-	-	-	-	-	-	-	0.10 ± 0.01	0.20 ± 0.02	-

GI_50_ (μΜ) of compounds tested *in vitro* for their antileishmanial activity against *L. donovani* promastigotes and intracellular amastigotes, and their cytotoxicity against murine macrophages J774.1. GI_50_ (μΜ) of the controls Amphotericin B, 6-BIO and 5-Me-6-BIO is also shown. Inhibitory activity was evaluated using the Alamar blue assay, as previously described. GI_50_ values were determined from dose–response curves via linear interpolation.

Indirubin analogues possessing halogeno groups (Br, CF_3_) in position 7 had no significant effect on *L. donovani* promastigote (GI_50_ > 3 μΜ) and intracellular amastigote growth (GI_50_ > 3 μΜ) (Table 1). On the contrary, the 3′- amino chain substituted 6BIO analogues **11–17** displayed antileishmanial activity against promastigotes at the low micromolar or nanomolar range (GI_50_: 0.65-1.50 μΜ). For these compounds, the intracellular amastigote assay was performed. Notably, the GI_50_ values obtained (GI_50_: 0.59-2.44 μΜ) were in the same range with the previously reported values of 6BIO and 5-Me-6-BIO [13]. The analogues **11–15** shown in Table 1 inhibited the *L. donovani* promastigotes and intracellular amastigotes equally well. Derivatives **16** and **17** inhibited the promastigotes with a GI_50_ of 0.76 ± 0.03 μΜ and 0.76 ± 0.04 μΜ respectively, but their inhibitory effects against intracellular amastigotes were slightly lower (GI_50_: 1.54 ± 0.11 μΜ and 1.22 ± 0.06 μΜ respectively). Interestingly, compounds **11** and **17** displayed a good selectivity index (>8) over the murine macrophagic cell-line J774.1, which was used to assess indirubin cytotoxicity (Table 1).

Next, we selected the indirubin analogues with antileishmanial activity (**11**–**17**) to evaluate their inhibitory effect towards *L*GSK-3 and *L*CRK3 kinases. In addition, to confirm that the selection of the compounds was unbiased, we selected several indirubins with no antileishmanial activity (compounds **2**, **27**, **30**, **33**) to perform the kinase assay. The parasitic kinases (*L*GSK-3 and *L*CRK3) were purified from homologous expression systems as previously described [13,18]. The Km values of both kinases for ATP and their respective substrate were in accordance to those previously reported [13]. The IC_50_ values were calculated from the dose–response curves. 6BIO and 5-Me-6-BIO [13] were tested in parallel and were used as reference controls. Interestingly, results obtained using *in vitro* assays against *L*GSK-3 and *L*CRK3 kinases, showed that the 6BIO analogues substituted with a piperazine or pyrrolidine ring at position 3′shifted the 6BIO selectivity towards *L*GSK-3 (Table 2). On the other hand, the selected analogues with no antileishmanial activity (compounds **2**, **27**, **30**, **33**), did not display any relevant activity towards *L*GSK-3 or *L*CRK3 (IC_50_ > 3.33 μΜ) (data not shown).

**Table 2:** **Inhibition of** ***L. donovani L*** **GSK-3 and** ***L. mexicana L*** **CRK3 by compounds 11–17**

	**IC**_**50**_**(μM)**	
**Compounds**	***L*** **GSK-3**	***L*** **CRK3**
**6-BIO**	0.15 ± 0.06	0.02 ± 0.01
**5-Me-6-BIO**	0.09 ± 0.02	0.65 ± 0.09
**11**	0.10 ± 0.07	>3.33
**12**	0.20 ± 0.08	0.99 ± 0.19
**13**	1.65 ± 0.17	>3.33
**14**	1.95 ± 0.11	>3.33
**15**	0.17 ± 0.25	0.14 ± 0.06
**16**	0.36 ± 0.18	>3.33
**17**	0.88 ± 0.26	>3.33

Inhibitory kinase activity of the controls 6BIO and 5-Me-6-BIO is also shown. IC_50_ values are expressed in μΜ.

Thus, herein (Table 2), we showed that the 7-fold selectivity displayed by 6BIO towards *L*CRK3 over *L*GSK-3 was shifted to *L*GSK-3 over *L*CRK3 selectivity upon the incorporation of a nitrogen-containing saturated ring in position 3' of the indirubin core. This type of substitution could enhance the affinity of the scaffold towards the hydrophilic sites of the binding pocket of *L*GSK-3, making these analogues more selective. As shown in Table 2, compound **11** which possesses only an additional piperazine ring substitution, displays a >33-fold degree of selectivity for *L*GSK-3 over *L*CRK3. These results indicate that the substitution of the oxime in position 3′ is an important feature for *L*GSK-3 inhibition. This finding is consistent with our previous observation that 6BIA inhibited *L*GSK-3 more potently [13].

**Figure 1:**
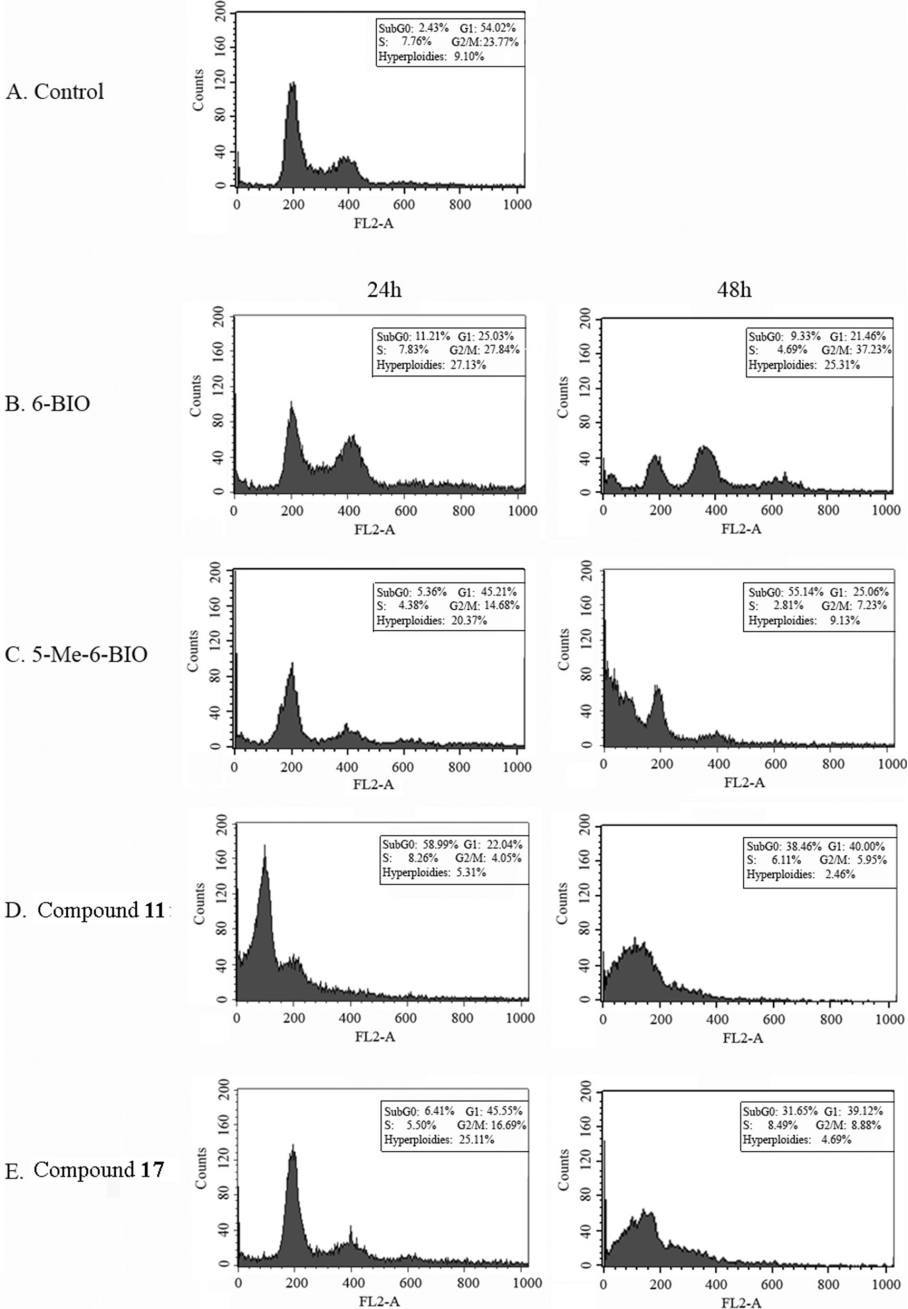
**Cell-cycle analysis of indirubin-treated cells by flow cytometry.** DNA content of parasites treated with vehicle only (**A**, control, DMSO < 0.1%) and parasites treated with GI_50_ concentration of compounds 11 **(D)**, 17 **(E)**. Compounds 6BIO **(B)** and 5-Me-6-BIO **(C)** were used as controls. The DNA content was measured by staining cells with propidium iodide (PI) and the cell-cycle status was analyzed by flow cytometry.

**Figure 2:**
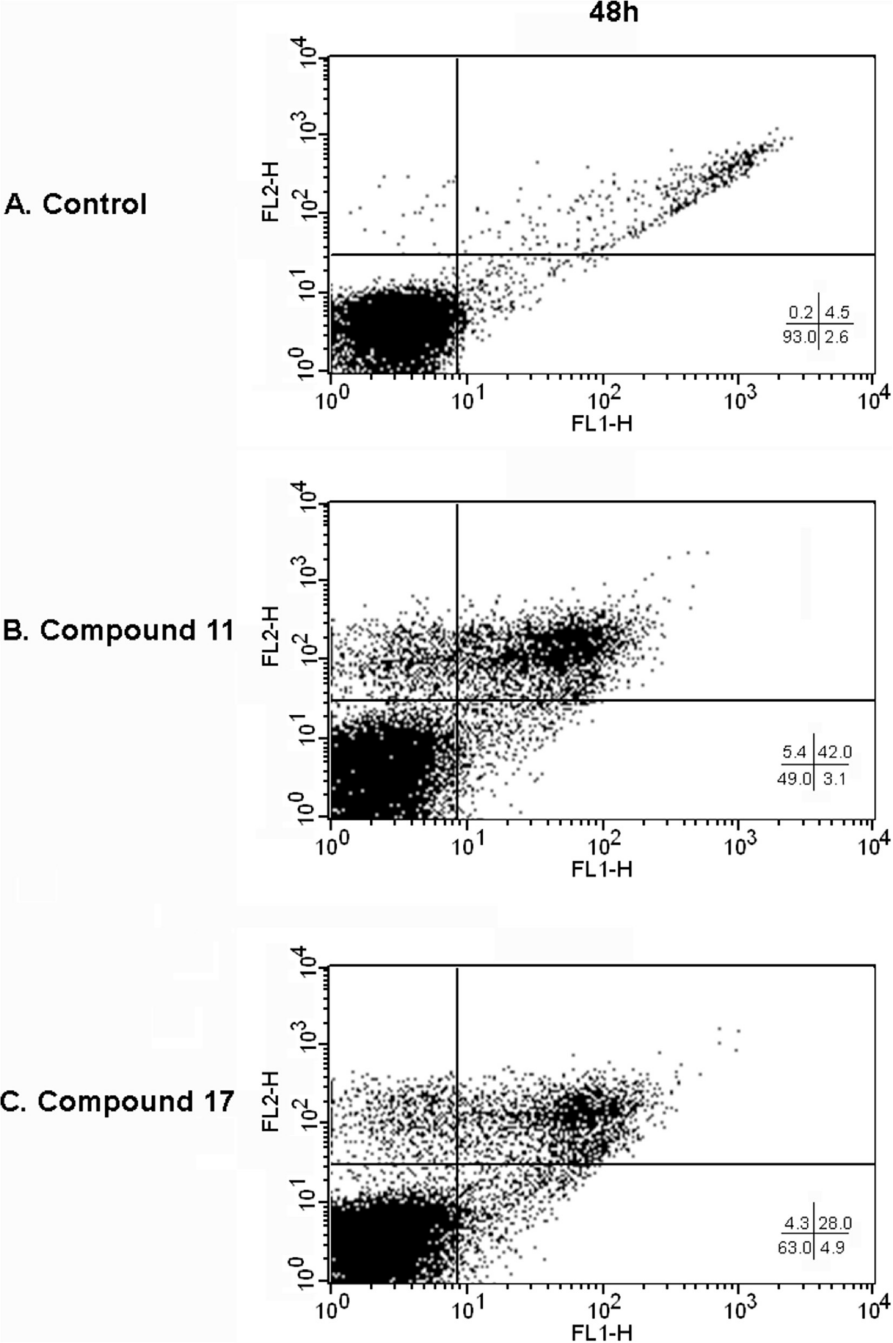
**Effect of indirubin analogues 11 and 17 on the cell-death of*L. donovani*promastigotes.** Effect of compounds 11 **(B)** and 17 **(C)** on the cell-death of *L. donovani* promastigotes, incubated with the GI_50_ of each compound for 48 hrs. Cells were stained with AnnexinV-FITC (horizontal axis, FL1-H) and propidium iodide PI (vertical axis, FL2-H) to assess membrane fluidity perturbations (AnnexinV staining) and membrane impermeability. The samples were then analyzed by flow cytometry. DMSO was used as negative control **(A)**.

In order to evaluate if the *in vitro* results of the shifted *L*GSK-3 over *L*CRK3 selectivity displayed by the analogues **11** and **17**, are also mirrored *in cellulo*, we have further examined their effect on the cell-cycle progression and the induction of parasitic cell-death. To this end, we have analyzed the cell-cycle progression in the *L. donovani* promastigotes after 24 and 48 hrs treatment with the compounds **11** and **17**. Indirubins were used at the GI_50_ concentration. 5-Me-6BIO and 6BIO, known to induce cell-cycle arrest in the G1 phase by inhibiting *L*GSK-3 and G2/M arrest by inhibiting *L*CRK3 respectively [13], were tested in parallel (Figure 1). Herein, *L. donovani* promastigotes treated with compounds 11 and 17 resulted in a rapid increase in the percentage of subG0/G1 cells (Figure 1). Cells treated with vehicle only [0.02% (v/v) DMSO], which were used as negative control, showed a normal distribution of the cell-cycle at all time-points studied (Figure 1). These results were in accordance with the distribution of the selective *L*GSK-3 inhibitor 5-Me-6-BIO, which served as a model compound for the association of *L*GSK-3 inhibition and the accumulation in subG0/G1 phase [13]. In addition, the contribution of **11** and **17** to the mode and timing of cell-death was tested. Thus, apoptotic-like cell-death was assessed by measuring membrane fluidity perturbation and membrane impermeability, until the late stages of the process. Double staining assay with AnnexinV-FITC and PI was used, which allows the differentiation between early apoptotic (AnnexinV-FITC positive), late apoptotic (AnnexinV-FITC and PI positive), necrotic (PI positive) and viable cells (unstained) [32-36]. Incubation of cells with 0.02% (v/v) DMSO showed negative staining for both AnnexinV and PI, as 93% of the cells were viable at all time-points (Figure 2). Compounds **11** and **17** caused a rapid increase of late apoptotic or necrotic cells after 48 hrs of incubation in the parasites (late apoptotic cells: 42% and 28% respectively, necrotic cells: 5.42% and 4.28% respectively). These results are in agreement with the mode of cell death caused by 5-Me-6-BIO and *L*GSK-3 inhibition as 48 hrs treatment with the compound resulted in an increase of the late apoptotic population [13]. On the other hand, parasites incubated with the *L*CRK3 inhibitor 6BIO, displayed an equal increase on early and late apoptotic cells [13], different to the cell-death pattern induced by compounds **11** and **17** displayed. In general, the above observations strengthen our results and suggest that the selectivity of the compounds **11** and **17** towards *L*GSK-3 is also mirrored in the parasite.

**Figure 3:**
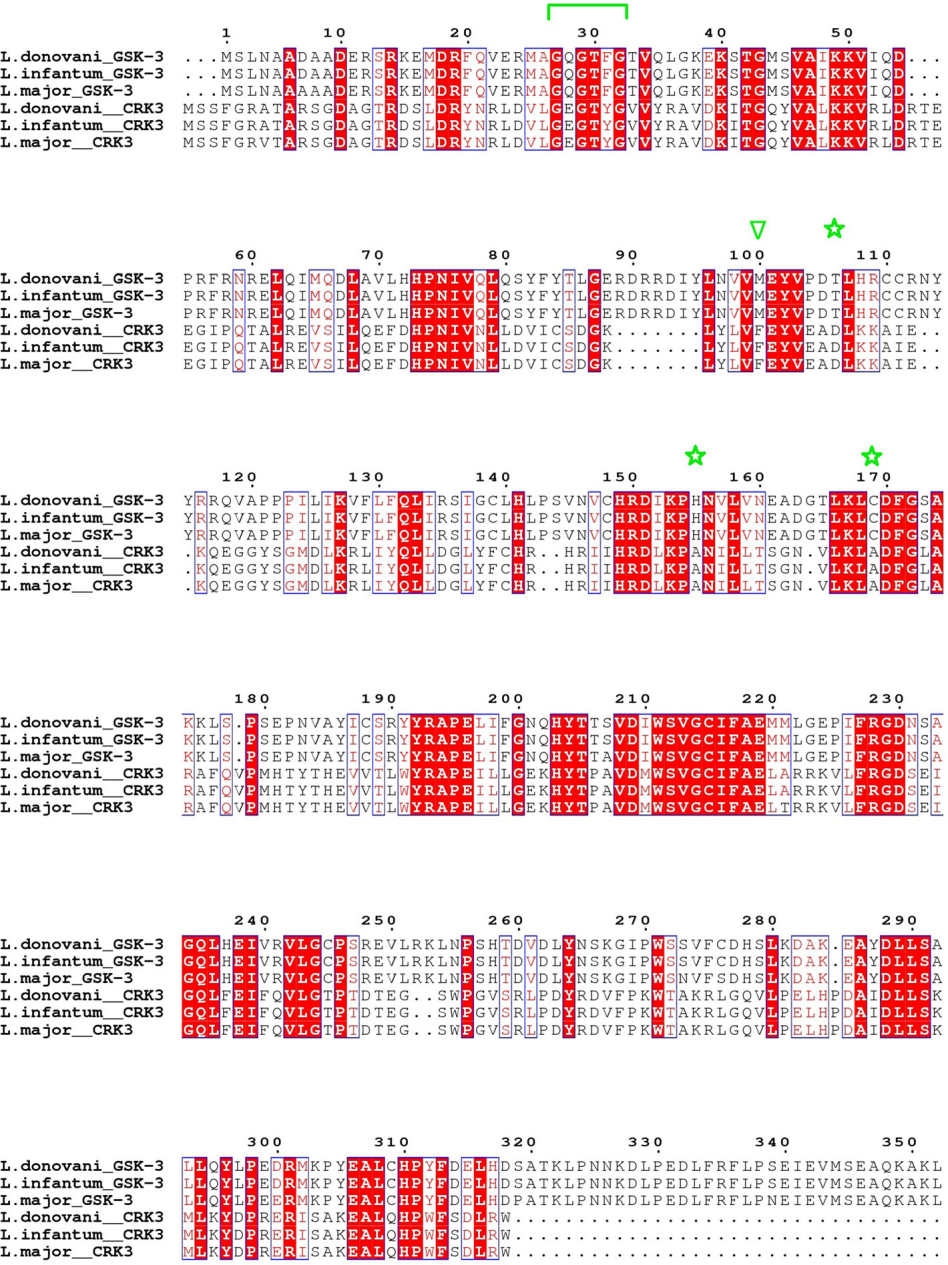
**Multiple sequence alignment of GSK-3 and CRK3 from three related*Leishmania*species.** The multiple sequence alignment depicts the high homology between GSK-3 and CRK3 of three *Leishmania* species, namely *L. donovani*, *L. infantum* and *L. major* (Uniprot codes: A6N857, A4HXQ3, Q4QE15 for GSK-3 and Q4K8Z1, A4ICT0, Q96526 for CRK3, respectively). The gatekeeper positions as well as the three non-conserved residues of the ribose sub-site are highlighted by green stars and the glycine-rich loop is marked by a green bracket. Alignment was performed using ClustalW with default settings and the depiction was prepared using ESPript software (http://espript.ibcp.fr) [37,38].

To better explain this shift towards *L*GSK-3, we performed a comparative structural analysis of *L*GSK-3 and *L*CRK3 homologue pairs (Figure 3) of three *Leishmania* species (*L. major*, *L. donovani*, *L. infantum*) [37,38]. Initial results showed that the active site residues demonstrated a high degree of sequence conservation within the group of studied homologues, with the most important differences being the replacement of the gatekeeper M100^*L*GSK-3^ to F99^*L*CRK3^. However, several variations were located at the vicinity of the binding pocket of the three *L*GSK-3/*L*CRK3 pairs and primarily on the glycine-rich loop and at the ribose and phosphate binding sub-sites of the kinases. In *L*GSK-3 the Gly-loop motifs is ^27^GQGTFG^32^ while in *L*CRK3 it is ^30^GEGTYG^35^. At the ribose site, T106^*L*GSK-3^ is replaced by D105^*L*CRK3^, while at the phosphate site H155^*L*GSK-3^ is replaced by A149^*L*CRK3^ and C169^*L*GSK-3^ by A162^*L*CRK3^. Those variations were identified as of possible importance with respect to the determination of the binding pocket hydration pattern. Moreover, it was suggested that differences in hydration could be indirectly induced through the sequence variations located at the glycine-loop by affecting its flexibility, which in turn might influence the interaction of the loop with the water molecules solvating the ribose and phosphate sub-sites.

**Figure 4:**
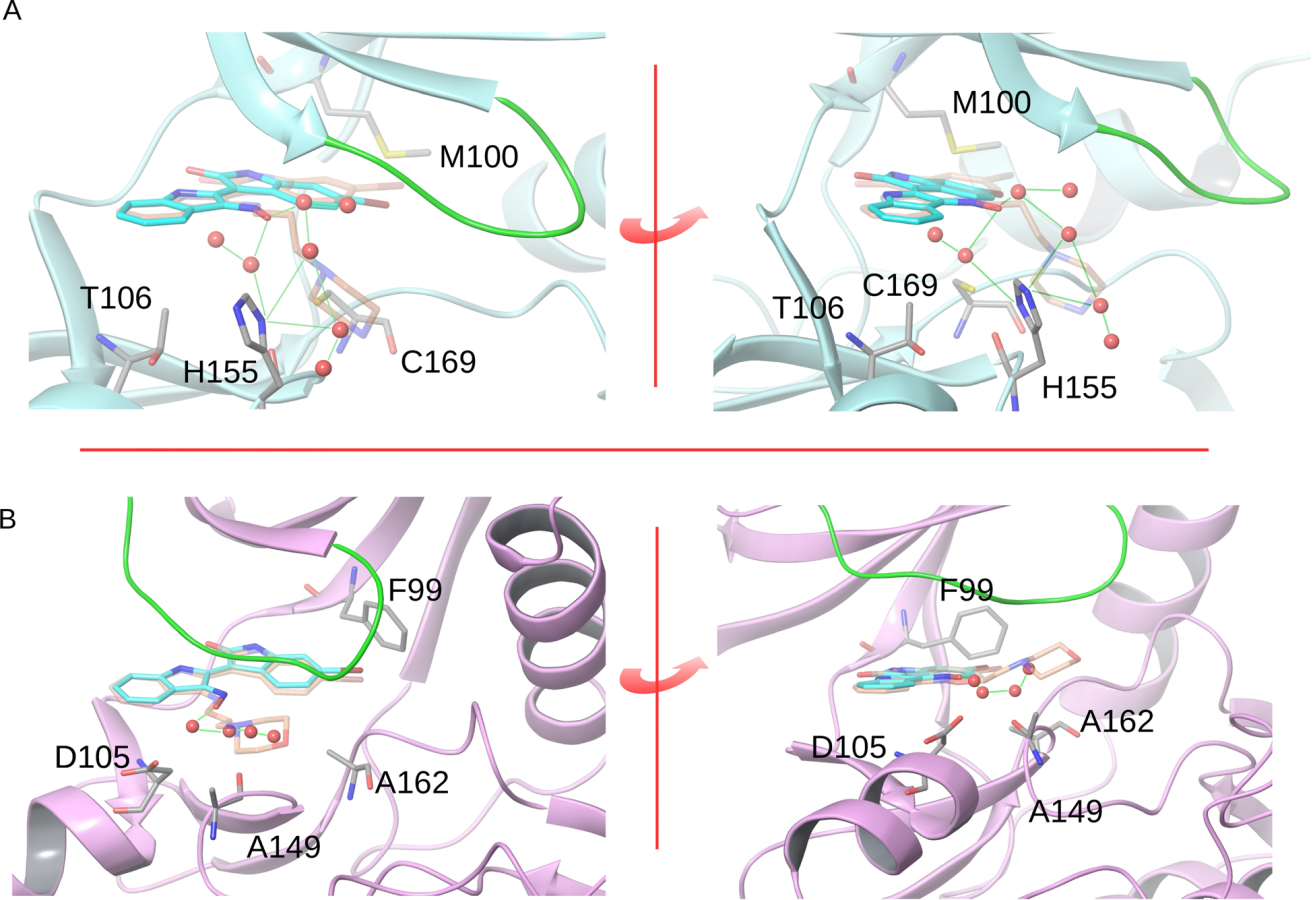
**Comparison of the binding pocket entrance hydration sites as mapped using Szmap algorithm between the leishmanial*L*GSK-3 and*L*CRK3.** Hydration sites with positive free energy as determined by Szmap computational analysis are depicted as red spheres in a ribbon representation of the protein. The hydration sites are connected with green lines depicting potential hydrogen bond networks that could be formed between them and neighboring residues. The depicted hydration sites potentially overlap with the bulky 3*'*-substituents of 6BIO analogues like those of compounds 11 and 17. Displacement and release of those water molecules by the compounds would favor binding affinity through the anticipated entropic gain. **A)** In *L*GSK-3, the indirubin substituent of position 3*'* occupies a region which overlaps with several unstable water molecules of the protein hydration shell. **B)** In *L*CRK3, the corresponding cluster of unstable water molecules is smaller and has a markedly lower overlap with the region occupied by the 3*'* substituent. As a result, binding affinity is not expected to be affected seriously by their displacement. In both figures, residues that differ between *L*GSK-3 and *L*CRK3 active sites are shown in stick representation and labelled (numbering based on *L. donovani* sequences). The ribbon fragments corresponding to the glycine-rich loop are colored green. The poses of analogues 11 and 17 are depicted as semi-transparent overlays on the 6BIO binding pose. For reasons of clarity and given that the poses of 11 and 17 are highly similar for each kinase, inhibitor 11 is depicted only in Figure 4A and inhibitor 17 in Figure 4B.

To assess that effect, the computational mapping of the hydration sites over the surface of the studied kinases was performed using Szmap algorithm [39,40]. Analysis of the hydration showed a pronounced difference on the predicted thermodynamic properties of water molecules, which would be expected to form the first hydration shell close to the ribose and phosphate binding sub-sites of each protein. More specifically, a cluster of waters of positive free energy is present at the aforementioned locations in the *L*GSK3 structures but not in the *L*CRK3 homologues (Figure 4). This cluster defines an area at which substitution of the solvent by polar ligand groups would favor binding affinity while replacement by hydrophobic groups would instead affect affinity in an unfavorable manner. Given the well-known impact of protein desolvation on binding affinity, it was hypothesized that compounds that bind the kinases through specific interactions accommodated at that specific part of the cavity could have a different affinity among the otherwise highly similar active sites of *L*GSK-3 and *L*CRK3.

Consistent with this hypothesis is the fact that the 6-bromo-3′-nitrogen-substituted analogues identified as lead molecules in the *L. donovani* growth inhibition assay, shifted their selectivity towards *L*GSK-3 over *L*CRK3 (**11–17**, Table 1). Those analogues could selectively target *L*GSK-3 by binding adjacent to the aforementioned region of the pocket, overlapping with the hydration sites of interest and thus gaining affinity due to the entropic effect of displacing relatively unstable water molecules from the kinase first hydration shell.

## Conclusions

In conclusion, the present study describes the screening of a small and diverse indirubin library for compounds with activity inhibiting the growth of leishmanial parasites and the identification of a class of 3′-bulky amino substituted indirubin analogues displaying inhibition and specificity on *L*GSK-3. Among these, derivatives **11** and **17** exerted a very good antileishmanial activity (GI_50_ < 0.76 μΜ in *L. donovani* promastigotes and GI_50_ < 1.22 μΜ in *L. donovani* intracellular amastigotes), and a good selectivity index (>8). Compound **11** possessed the highest *L*GSK-3 over *L*CRK3 selectivity index (>33-fold). Thus, these compounds are considered as an initial scaffold for the design of *L*GSK-3 selective inhibitors and therefore for the design of potent antileishmanial agents.

## Competing interests

The authors declare that they have no conflict of interest.

## Authors’ contributions

KS, A-LS and DS conceived and designed the study. AE, NG-K, KV, MK and DS carried out the experiments and analyzed the data. VM and EM carried out the computational analysis and analyzed the data. AE, A-LS, KS, DS, NG, VM and KV drafted and revised the manuscript. AA, EM provided analysis tools and/or contributed to the critical revision of the manuscript. All authors read and approved the final version of the manuscript.
